# Similarities between the responses to ANT‐DBS and prior VNS in refractory epilepsy

**DOI:** 10.1002/brb3.983

**Published:** 2018-05-08

**Authors:** Toni Kulju, Joonas Haapasalo, Kai Lehtimäki, Sirpa Rainesalo, Jukka Peltola

**Affiliations:** ^1^ Department of Neurosciences and Rehabilitation Tampere University Hospital Tampere Finland; ^2^ Faculty of Medicine University of Tampere Tampere Finland

**Keywords:** deep brain stimulation, epilepsy, follow‐up, seizure, vagus nerve stimulation

## Abstract

**Objectives:**

Neurostimulation has offered new treatment options in refractory epilepsy, first with vagus nerve stimulation (VNS) and more recently with deep brain stimulation (DBS). There is a lack of previous detailed data assessing the relationship between VNS and ANT‐DBS. The aim of this study was to investigate the potential correlation between therapeutic responses to VNS and ANT‐DBS.

**Materials and Methods:**

A total of 11 patients with previous VNS therapy underwent ANT‐DBS implantation. Monthly seizure counts starting from baseline before VNS extending to long‐term DBS treatment were analyzed. The reasons for VNS discontinuation were assessed.

**Results:**

Altogether in 10 of 11 patients, the response to VNS seemed to be similar to the response to DBS therapy. Progressive response to VNS was likely to correlate with a progressive response to DBS in three of three patients. Partial response to VNS was associated with a fluctuating response pattern to DBS in two patients. Five of six nonresponders to VNS were also nonresponders to DBS. One of the VNS nonresponders obtained progressive response to DBS.

**Conclusions:**

This is the first study to evaluate in detail the effect of both VNS and ANT‐DBS in refractory epilepsy patients. There is a putative association between VNS and DBS responses suggesting the need for further studies.

## 1 INTRODUCTION

Patients with refractory epilepsy comprise approximately 30% of all patients with epilepsy (Kwan & Brodie, 2000). Resective surgery is the treatment of choice for this patient group with focal epilepsy, but only 10‐30% of patients are eventually amenable for surgery. Optimizing the pharmacological treatment can make some of these patients seizure free (Liimatainen, Raitanen, Ylinen, Peltola, & Peltola, 2008), but possibilities for major improvement with antiepileptic drug (AED) therapy are limited. Neurostimulation has offered new treatment options in refractory epilepsy, first with vagus nerve stimulation (VNS) and later with deep brain stimulation (DBS) of the anterior nucleus of the thalamus (ANT).

VNS delivers an electrical current to the 10th cranial nerve via electrode, wrapped around surgically the exposed vagal nerve. Currently, VNS devices are being implanted in patients with refractory seizures who cannot have resective surgery or who have had surgery with poor results. Moreover, many of these patients have been treated with several antiepileptic drugs before receiving VNS implants (Ben‐Menachem, 2002). The biological mechanisms causing the effects of vagus nerve stimulation are still not fully understood (Roosevelt, Smith, Clough, Jensen, & Browning, 2006). VNS has been reported to reduce seizure frequencies by more than 50% in a group of patients with refractory epilepsy ranging from 30% (Ryvlin et al., 2014) to 50% (Cukiert, 2015).

Deep brain stimulation is a promising treatment choice for refractory focal epilepsy showing sustaining efficacy and safety (Salanova et al., 2015). DBS delivers currents directly to the ANT via electrodes implanted using stereotactic neurosurgical technique. The most optimal stimulation site is not unambiguously defined at this moment, and detailed anatomical variation of electrode location may have an effect on the outcome (Lehtimäki et al., 2016; Möttönen et al., 2015).

There is some evidence suggesting common pathways between VNS and ANT‐DBS therapy. In VNS, there are synaptic connections from the nucleus tractus solitarius to higher centers in the brain including thalamus (Rutecki, 1990). Furthermore, positron emission tomography (PET) and functional magnetic resonance imaging (fMRI) of the effects of VNS in human beings have confirmed the influence of the vagus nerve on higher brain structures (Ko et al., 1996). These data suggest that the thalamus is consistently involved in VNS therapy.

The scientific basis for rational selection between different neuromodulation therapies is lacking. First, in the SANTE trial there was a subset of patients with previous VNS and/or resective surgery, but no predictive association with DBS was reported (Fisher et al., 2010). Second, there are no follow‐up studies evaluating the modification from VNS to DBS (or vice versa). Third, the comparison between the efficacies of these treatment modalities has been challenging as there are only case reports about patients with both VNS and DBS implanted (Franzini et al., 2009).

To our knowledge, this is the first study comparing in detail the long‐term results of VNS and DBS therapy. Eleven patients with previous VNS therapy later underwent ANT‐DBS. Monthly seizure counts from the baseline before VNS to long‐term DBS treatment were analyzed.

## 2 MATERIALS AND METHODS

A total of 11 patients with previous VNS were implanted with ANT‐DBS in Tampere University Hospital, Tampere, Finland, for refractory epilepsy. The VNS surgeries were performed in 2005‐2011 and DBS in 2010‐2013. All patients had been evaluated using inpatient video‐EEG (electroencephalography) telemetry, 18‐F‐FDG‐PET (fluorodeoxyglucose–positron emission tomography), and 3T MRI (3 Tesla magnetic resonance imaging) to identify potential epileptogenic zone/epileptic syndrome and evaluated for resective surgery. Clinical features of the patients are summarized in Table 1. The Study Plan was approved by the Ethical Committee of Tampere University Hospital, Tampere, Finland.

**Table 1:** Patient characteristics

No.	Sex	Age At VNS implant	MRI	Etiology	Seizure onset zone	Duration of VNS therapy	VNS responder	Reason for VNS discontinuation	DBS responder
1	M	18	Normal	Encephalitis	Multifocal	4 years 2 month	Yes, progressive	Battery depletion	Yes, progressive
2	F	17	Fronto‐parietal bilateral gliosis	Encephalitis	Multifocal	4 years	Yes, progressive	Not effective enough	Yes, progressive
3	M	34	Bilateral perisylvian polymicrogyria	CD	Multifocal	5 years 3 month	Yes, progressive	High impedance	Yes, progressive
4	F	20	Bilateral perisylvian polymicrogyria	CD	Multifocal	5 years 1 month	Yes, partial	Lack of sustained efficacy	Yes, partial
5	M	24	Occipital bilateral heterotopia	CD	Multifocal	5 years 2 month	Yes, partial	Lack of sustained efficacy	Yes, partial
6	M	49	Normal	Unknown	Left temporal	6 years 6 month + 9 month and still ON	No	DBS implant, VNS not removed	No
7	M	42	Bilateral perisylvian polymicrogyria	CD	Multifocal	3 years 4 month	No	Lack of efficacy	No
8	M	19	Normal	Encephalitis	Left frontotemporal	3 years 8 month	No	Lack of efficacy	No
9	M	17	Normal	Encephalitis	Multifocal	2 years 8 month (2 m break)	No	Lack of efficacy	No
10	M	38	Normal	Unknown	Frontal lobe, side unknown	4 years 3 month + 1 years 7 month	No	Lack of efficacy	No
11	F	29	Right hemi‐ megalencephaly	CD	Right frontotemporal	4 years 9 month	No	Lack of efficacy	Yes, progressive

VNS was implanted microsurgically by exposing carotid sheath and the left vagus nerve, located medial to the jugular vein. A coil electrode (Cyberonics, USA) was wrapped around the vagus nerve, and the lead was fixed utilizing silicon anchors as recommended by the manufacturer. An internal pulse generator (Cyberonics) was implanted to the subcutaneous upper chest. From 10 of 11 patients, the VNS device was surgically removed before the implantation of DBS. DBS leads (3389, Medtronic) were stereotactically implanted bilaterally under general anesthesia using visual targeting based on 3T MRI STIR (Short Tau Inversion Recovery) images (Lehtimäki et al., 2016; Möttönen et al., 2015). An internal pulse generator (Activa PC, Medtronic, USA) was implanted to the subcutaneous upper chest. DBS was started within few days after surgery.

The period of effective VNS therapy was defined as a successful delivery of significant therapeutic currents. The effective VNS therapy could be terminated by turning the current OFF, depletion of the battery, or with a high impedance situation. The reasons for VNS discontinuation were re‐evaluated. The varying VNS stimulation parameters were programmed individually to reach the best clinical outcome, the mean VNS settings at the end of the follow‐up being the following: output current 2.1 mA, cycle 28.5 s ON / 66.5 s OFF, frequency 30 Hz, and pulse width 500 μs. The mean DBS settings at the end of the follow‐up were the following: voltage 5.8 V (left), 5.3 V (right), frequency 147.3 Hz, pulse width 124.5 μs, and cycle 1 min ON / 5 min OFF. The programming of the DBS electrodes was planned according to MRI imaging.

The number of seizures during the year prior to VNS operation and afterward was evaluated from the patient records retrospectively for the majority of patients. For five patients, the original seizure diaries were possible to obtain for seizure counting for the entire time period. The response to the stimulation is considered as “yes” if there is a decrease of more than 50% in the total number of seizures (6 months average seizure count in any time point with effective stimulation) compared to the baseline (12 months average seizure count before VNS/DBS implantation). A progressive VNS responder is defined as a patient with continuous progressive decline in seizure frequency during effective VNS therapy. A partial VNS responder is defined as a patient with an initial >50% decrease in seizure frequency but with fluctuating seizure count over long term. A progressive DBS responder is defined as a patient with continuous progressive declination in seizure frequency during effective DBS therapy. A partial DBS responder is defined as initial >50% decrease in seizure frequency but with fluctuating seizure count over long term. A nonresponder to VNS or DBS is defined as less than 50% decrease in seizure frequency over the course of neurostimulation therapy. The patients having “partial response” cannot be considered as true responders as the effect does not sustain. We also briefly assessed the changes in drug treatment in every patient.

## 3 RESULTS

**Figure 1:**
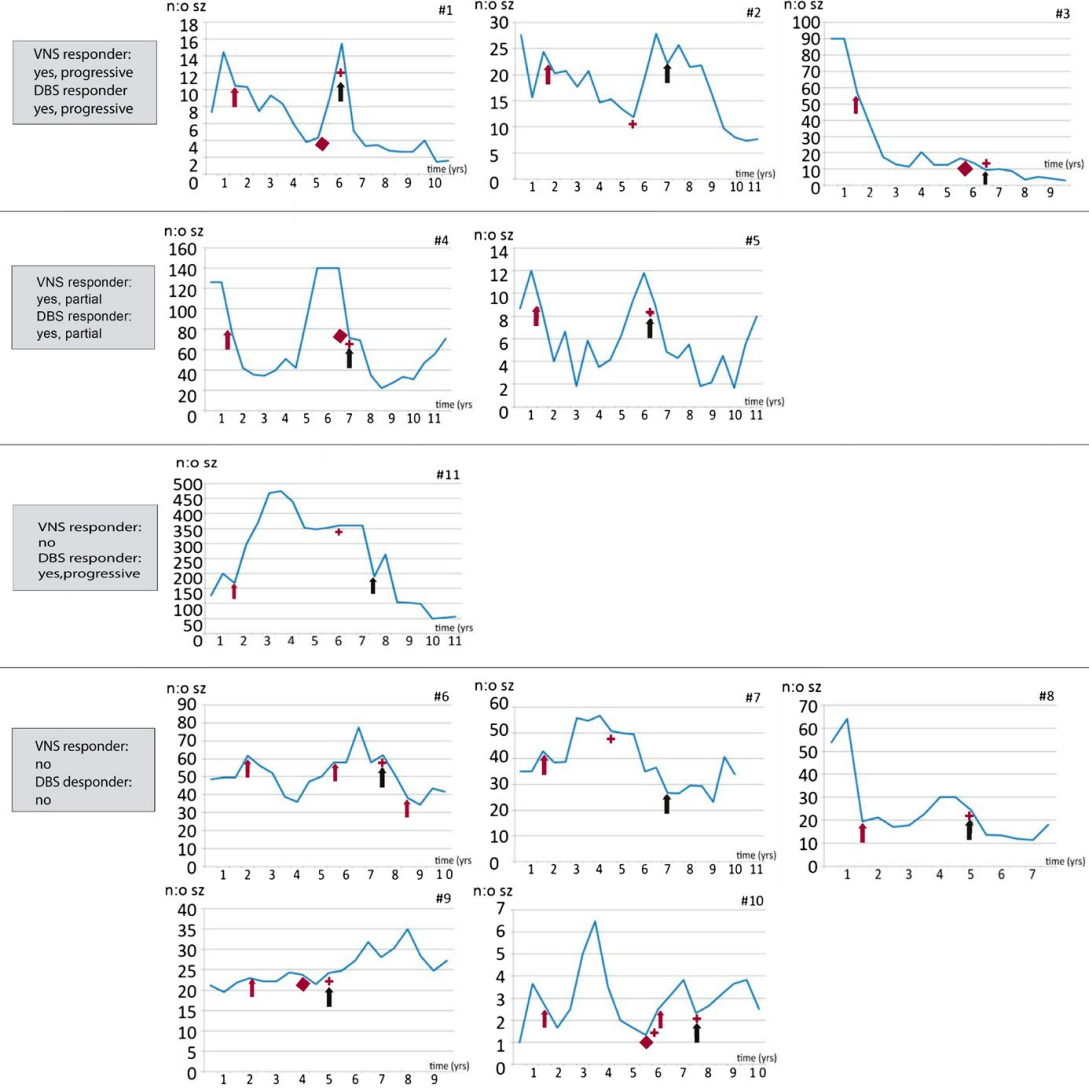
Mean monthly seizure count in six‐month intervals. Legend: red arrow: VNS ON, red star: VNS OFF, red diamond: VNS high impedance in patients 3, 4, and 9, and battery depletion in patients 1 and 10, black arrow: DBS ON. Note: patient #6, second red arrow represents VNS battery change

Altogether in 10 patients of 11 (91%), VNS response was similar to the response pattern to DBS therapy. Three of 11 patients were stable responders to VNS therapy. All these patients showed also progressive response to DBS therapy. Two patients had an initial response to VNS therapy that was not sustained over the course of years. All these patients demonstrated a partial response to DBS therapy. Six patients did not have satisfying effects by VNS based on the total number of seizures, and five of these patients did not have a response neither to DBS therapy. Only in one nonresponder to VNS therapy, there was a progressive response to DBS therapy. The details of seizure counts are shown in Figure 1 and patient characteristics in Table 1.

At the time of the VNS initiation, three of the patients were on AED monotherapy, one patient on two AEDs, five patients on three AEDs, and two patients on four AEDs, 2.54 AEDs on average. Subsequently, while proceeding to DBS therapy, three patients were on AED monotherapy, none of them on two AEDs, six patients on three AEDs, one patient on four AEDs, and one patient on five AEDs. At the end of follow‐up, they were on 2.91 AEDs on average; one patient was on AED monotherapy, two patients on two AEDs, five patients on three AEDs, and three patients on four AEDs. During the VNS therapy, seven new AED introductions were enacted, along with the five increases in dosage, six decreases in dosage, and five discontinuations. During DBS therapy, seven new AED introductions, five increases, three decreases, and six discontinuations were executed. In one of the patients, no AED changes were made. The alterations in AED regimen enacted during the follow‐up are presented in Table 2. In the group of nonresponders to both therapies, one AED introduction was made between the neuromodulation therapies, causing the contradiction in total number of AEDs in the table. None of the responses to VNS or DBS are explained by the AED changes. The stimulation parameters were adjusted in every patient according to a similar protocol, with similar goal settings. We re‐evaluated the stimulation setting histories and did not find any differences in the settings between the responders and nonresponders.

**Table 2:** Antiepileptic drug schedule alterations during the follow‐up

Responder status to VNS & DBS therapies	Initiative AEDs (average count)	AED changes during VNS therapy	AED count on DBS implantation (average count)	AED changes during DBS therapy	AED count at the end of the follow‐up (average count)
Progressive & progressive (*n* = 3)	3	2 introductions 3 decreases 1 discontinuation	3.33	1 introduction 1 decrease 1 discontinuation	3.33
Partial & partial (*n* = 2)	2	1 introduction 3 increases 1 discontinuation	2	1 introduction 2 increases 1 decrease	2.5
No & no (*n* = 5)	2.4	1 introduction 2 increases 2 decreases 2 discontinuations	2.2	3 introductions 2 increases 1 decrease 2 discontinuations	2.6
No & progressive (*n* = 1)	3	3 introductions 1 decrease 1 discontinuation	5	2 introductions 1 increase 3 discontinuations	4

## 4 DISCUSSION

According to our descriptive study, the response to ANT‐DBS therapy seems to be clinically associated with the response to previous trial of VNS therapy; if a patient had a partial or progressive positive effect of VNS, the ANT‐DBS effect also showed same feature. If the patient was not responding to VNS therapy at all, the chances for a stable DBS response were reduced. Interestingly, this study provides for a first time a long‐term follow‐up data for more than 10 years of patients with both VNS and DBS therapies. The follow‐up data of the SANTE trial have been published recently (Salanova et al., 2015): The median seizure reduction with ANT‐DBS compared with baseline for patients previously tried with VNS was 69% in five years, whereas the seizure reduction without prior VNS was also 69%. The reasons for VNS discontinuation were not reported. Therefore, their results differ from ours, as they did not report any similarities between the responses to VNS and ANT‐DBS therapies.

This discrepancy between our results is most likely due to the different nature of the SANTE trial patient population and our study population. The patients in the SANTE trial with previous VNS therapy were classified as nonresponders; however, the reasons for VNS explantation may vary including scar formation with impedance problems, battery depletion, or dissatisfaction with obtained response, and the study might contain a group of patients with heterogeneous responses ranging from total nonresponders to partial responders. The patients for clinical trials are selected by more rigorous assessments than it is the case in everyday clinical practice. Most likely, in the SANTE trial, patients with a good response to VNS were not included. In our study population, some good VNS responders were indeed changed to DBS therapy, owing to the fact that at the time of the decision to proceed to DBS, the full effect of VNS was not acknowledged. Also, it has to be taken into consideration that our patients were not fully satisfied with the VNS response and wanted to have it better in spite of being responder to VNS according to conventional evaluation.

Our study demonstrates that the reasons for discontinuing VNS treatment can be variable. Some patients did not have any effect of VNS on seizure frequency, therefore forming one distinct group. Most of the patients with VNS treatment showed some response to the treatment. Furthermore, one patient group had an initial response fulfilling the traditional criteria for responder, but later lost this response despite continuous effective VNS treatment forming a group of fluctuating partial responders. This group cannot be considered as real responders, however, but form an interesting group of patients as they seem to respond to both VNS and ANT‐DBS in a similar way, although the effect could also be explained by the fluctuating nature of the disease. A third group demonstrated a progressive decrease in seizure frequency until VNS therapy was either intentionally or unintentionally (battery depletion) terminated, or the therapy was no longer effective due to high impedance situation. There was a high impedance situation in three of 11 patients in our study group, which is quite exceptional and does not present the usual prevalence within our center. In high impedance situations, we tend to consider other treatment options along with the lead revision surgery. Another option for some of our patients could have been re‐implantation of the VNS electrodes or simply battery replacement, instead of commencing the DBS therapy. In clinical setting, some VNS responders were considered as nonresponders, which was realized afterward in the retrospective analysis. There is also an option of re‐introducing VNS therapy in combination with ANT‐DBS therapy for a possibility of additive efficacy. These findings highlight the importance of precise and detailed information about seizures, for example, seizure diaries and careful patient follow‐up, performed in our study in a single epilepsy center and by one epileptologist (JP). Furthermore, before altering the treatment method from VNS to DBS (or vice versa), a long‐term follow‐up of different seizure types and their frequency should be carefully assessed.

Along with the neurostimulation treatment, the patients were treated with antiepileptic drugs in accordance with standard clinical practice. Within the patients responding to VNS and DBS therapies, the AED regimen alterations do not seem to cause significant effects on seizure frequencies, even though the total AED amount being slightly increased during the follow‐up period. None of the responders to VNS or DBS were so because of the AED changes.

There are data suggesting commonalities in VNS and ANT‐DBS treatments. VNS increased cerebral blood flow (CBF) to the right thalamus among other structures such as the right posterior temporal cortex (Ko et al., 1996). Additionally, in PET studies blood flow was increased to the inferior cerebellum, hypothalamus, and thalamus and decreased in the areas of the hippocampus, amygdala, and posterior cingulate gyrus during VNS (Henry et al., 1998). In a subsequent study, increased right and left thalamic CBF correlated with decreased seizures suggesting that increased thalamic synaptic activities probably mediate some anticonvulsant effects of VNS (Henry et al., 1999). The main conclusions from these studies are that the thalamus is consistently involved in VNS therapy (Ben‐Menachem, 2002), which lend support to the hypothesis that DBS stimulation of the ANT with prominent connections with limbic circuitry affects similar structures with VNS. One might speculate that the similar responses to VNS and DBS therapy in our patient population might be partly explained by this neurobiological concept.

On the one hand, the main limitation of our study is the small number of patients with both VNS and DBS treatments limiting the possibilities for statistical analysis. On the other hand, all previous ANT‐DBS studies with the exception of the SANTE (Salanova et al., 2015) trial comprise similar numbers of patients. We also provide long‐term follow‐up data for more than seven years for each patient. Another significant weakness of our study is that the data are collected retrospectively and the trial is unblinded and nonrandomized, therefore increasing the risk of bias in the results. Moreover, some segment of the response might be fallacious due to the fluctuating nature of the disease.

As a conclusion, this is the first study to evaluate in detail the effect of both VNS and ANT‐DBS therapies in refractory epilepsy patients. Our study provides some provisional data suggesting an interesting relationship between responses to two modalities of neurostimulation. The main feature of our study is to form a hypothesis for further analysis. Much information on the detailed VNS response in patients with subsequent ANT‐DBS therapy is needed to assess the definitive significance of this putative association.

## CONFLICT OF INTEREST AND SOURCES OF FUNDING

Joonas Haapasalo has received support for travel to congresses from Medtronic and Stryker. Kai Lehtimäki has received consultation fees and speaker honoraria from Medtronic and Abbot (former St. Jude Medical). Sirpa Rainesalo has received speaker honoraria from Fenno Medical, Orion Pharma, and UCB. Jukka Peltola has participated in clinical trials for Eisai, UCB, and Bial; received research grants from Eisai, Medtronic, UCB, and Cyberonics; received speaker honoraria from Cyberonics, Eisai, Medtronic, Orion Pharma, and UCB; received support for travel to congresses from Cyberonics, Eisai, Medtronic, and UCB; and participated in advisory boards for Cyberonics, Eisai, Medtronic, UCB, and Pfizer. The remaining authors have no conflict of interests.

## References

[brb3983-bib-0001] Ben‐Menachem, E. (2002). Vagus‐nerve stimulation for the treatment of epilepsy. The Lancet Neurology, 1, 477–482. https://doi.org/10.1016/S1474-4422(02)00220-X 12849332 10.1016/s1474-4422(02)00220-x

[brb3983-bib-0002] Cukiert, A. (2015). Vagus nerve stimulation for epilepsy: An evidence‐based approach. Progress in Neurological Surgery, 29, 39–52. https://doi.org/10.1159/issn.0079-6492 26393531 10.1159/000434654

[brb3983-bib-0003] Fisher, R. , Salanova, V. , Witt, T. , Worth, R. , Henry, T. , Gross, R. , … SANTE Study Group . Electrical stimulation of the anterior nucleus of thalamus for treatment of refractory epilepsy. Epilepsia (2010). 89, 9–908. 10.1111/j.1528-1167.2010.02536.x 20331461

[brb3983-bib-0004] Franzini, A. , Messina, G. , Leone, M. , Cecchini, A. P. , Broggi, G. , & Bussone, G. (2009). Feasibility of simultaneous vagal nerve and deep brain stimulation in chronic cluster headache: Case report and considerations. Neurology Science, 30(suppl 1), 137–139. https://doi.org/10.1007/s10072-009-0076-0 10.1007/s10072-009-0076-0 19415445

[brb3983-bib-0005] Henry, T. R. , Bakay, R. A. , Votaw, J. R. , Pennell, P. B. , Epstein, C. M. , Faber, T. L. , … Hoffman, J. M. (1998). Blood flow alterations induced by therapeutic vagus nerve stimulation in partial epilepsy 1: Acute effects at high and low levels of stimulation. Epilepsia, 39, 983–989. https://doi.org/10.1111/j.1528-1157.1998.tb01448.x 9738678 10.1111/j.1528-1157.1998.tb01448.x

[brb3983-bib-0006] Henry, T. R. , Votaw, J. R. , Pennell, P. B. , Epstein, C. M. , Bakay, R. A. , Faber, T. L. , … Hoffman, J. M. (1999). Acute blood flow changes and efficacy of vagus nerve stimulation in partial epilepsy. Neurology, 52, 1166–1173. https://doi.org/10.1212/WNL.52.6.1166 10214738 10.1212/wnl.52.6.1166

[brb3983-bib-0007] Ko, D. , Heck, C. , Grafton, S. , Apuzzo, M. L. , Couldwell, W. T. , Chen, T. , … DeGiorgio, C. M. (1996). Vagus nerve stimulation activates central nervous system structures in epileptic patients during PET H215O blood flow imaging. Neurosurgery, 39, 426–430. https://doi.org/10.1097/00006123-199608000-00061 8832691 10.1097/00006123-199608000-00061

[brb3983-bib-0008] Kwan, P. , & Brodie, M. J. (2000). Early identification of refractory epilepsy. New England Journal of Medicine, 342, 314–319. https://doi.org/10.1056/NEJM200002033420503 10660394 10.1056/NEJM200002033420503

[brb3983-bib-0009] Lehtimäki, K. , Möttönen, T. , Järventausta, K. , Katisko, J. , Tähtinen, T. , Haapasalo, J. , … Peltola, J. (2016). Outcome based definition of the anterior thalamic deep brain stimulation target in refractory epilepsy. Brain Stimulation, 9, 268–275. https://doi.org/10.1016/j.brs.2015.09.014 26680105 10.1016/j.brs.2015.09.014

[brb3983-bib-0010] Liimatainen, S. P. , Raitanen, J. A. , Ylinen, A. M. , Peltola, M. A. , & Peltola, J. T. (2008). The benefit of active drug trials is dependent on aetiology in refractory focal epilepsy. Journal of Neurology, Neurosurgery and Psychiatry, 79, 808–812. https://doi.org/10.1136/jnnp.2007.132811 10.1136/jnnp.2007.132811 17991701

[brb3983-bib-0011] Möttönen, T. , Katisko, J. , Haapasalo, J. , Tähtinen, T. , Kiekara, T. , Kähärä V, P. , … Lehtimäki, K. (2015). Defining the anterior nucleus of the thalamus (ANT) as a deep brain stimulation target in refractory epilepsy: Delineation using 3 T MRI and intraoperative microelectrode recording. NeuroImage Clinical, 7, 823–829. https://doi.org/10.1016/j.nicl.2015.03.001 26082891 10.1016/j.nicl.2015.03.001 PMC4459042

[brb3983-bib-0012] Roosevelt, R. W. , Smith, D. C. , Clough, R. W. , Jensen, R. A. , & Browning, R. A. (2006). Increased extracellular concentrations of norepinephrine in cortex and hippocampus following vagus nerve stimulation in the rat. Brain Research, 1119, 124–132. https://doi.org/10.1016/j.brainres.2006.08.048 16962076 10.1016/j.brainres.2006.08.04 PMC1751174

[brb3983-bib-0013] Rutecki, P. (1990). Anatomical, physiological, and theoretical basis for the antiepileptic effect of vagus nerve stimulation. Epilepsia, 31(suppl 2), S1–S6. https://doi.org/10.1111/j.1528-1157.1990.tb05843.x 10.1111/j.1528-1157.1990.tb05843.x 2226360

[brb3983-bib-0014] Ryvlin, P. , Gilliam, F. G. , Nguyen, D. K. , Colicchio, G. , Iudice, A. , Tinuper, P. , … Perucca, E. (2014). The long‐term effect of vagus nerve stimulation on quality of life in patients with pharmacoresistant focal epilepsy: The PuLsE (Open Prospective Randomized Long‐term Effectiveness) trial. Epilepsia, 55, 893–900. https://doi.org/10.1111/epi.12611 24754318 10.1111/epi.12611 PMC4283995

[brb3983-bib-0015] Salanova, V. , Witt, T. , Worth, R. , Henry, T. R. , Gross, R. E. , Nazzaro, J. M. , … SANTE Study Group (2015). Long‐term efficacy and safety of thalamic stimulation for drug‐resistant partial epilepsy. Neurology, 84, 1017–1025. https://doi.org/10.1212/WNL.0000000000001334 25663221 10.1212/WNL.0000000000001334 PMC4352097

